# Characteristic Analysis of Featured Genes Associated With Stemness Indices in Colorectal Cancer

**DOI:** 10.3389/fmolb.2020.563922

**Published:** 2020-10-06

**Authors:** Yongqu Lu, Xin Zhou, Zhenzhen Liu, Wendong Wang, Fei Li, Wei Fu

**Affiliations:** Department of General Surgery, Peking University Third Hospital, Beijing, China

**Keywords:** biomarker, colorectal cancer, cancer stem cell, stemness index, WGCNA

## Abstract

Cancer stem cells (CSCs) with self-renewal play an important role in tumor initiation and progression and are associated with drug resistance in cancer therapy. Here, we investigated the characteristics of stem cell-related genes in colorectal cancer (CRC) based on datasets from The Cancer Genome Atlas (TCGA) and Oncomine. We found that the stemness indices were significantly overexpressed in CRC tissues and were associated with patient survival. Weighted gene co-expression network analysis (WGCNA) was performed to determine the modules of stemness and featured genes. Significant modules and 8 genes (*BUB1*, *BUB1B*, *CHEK1*, *DNA2*, *KIF23*, *MCM10*, *PLK4*, and *TTK*) were selected according to the inclusion criteria. Expression analyses of transcription and protein levels confirmed internal correlation and their relevance with the tumor. Functional analysis of these genes demonstrated their enrichment in pathways, including checkpoint, chromosomal region and protein serine/threonine kinase activity. These results suggested that the characteristics of the featured genes fit well with CRC pathology and could provide new strategies for individual prevention and treatment.

## Introduction

Cancer stem cells (CSCs) that fuel tumor hierarchies through minor self-renewal are believed to be dominant in many tumor types, with mechanisms similar to the protection of normal stem cells and its resistance to standardized chemotherapy and radiation treatment partly accounts for disease relapse after cancer treatment (Clevers, 2011). Studies have confirmed that CSCs can generate tumors efficiently upon xenotransplantation without modified stimuli in the environment (Batlle and Clevers, 2017). Programs that focus on eliminating tumor cells have been launched, including proposed therapeutic strategies to interfere with CSCs and some conceptions have been progressively verified in different cancers from hematological malignancies to solid tumors (Harris et al., 2012; Boumahdi et al., 2014). The idea of anti-CSC therapy, aimed at key signaling pathways, epigenetic alterations or other CSC-targeting approaches, benefits from the exploration of biological markers in great depth (Nervi et al., 2015; Takebe et al., 2015). CSC-associated signals and epigenetic mechanisms, as well as regulation from specific microenvironments, contribute to depict stemness potential and classify the functional characterization of CSCs that are isolated from the genetic background of tumor cells (Maccalli and De Maria, 2015; Nassar and Blanpain, 2016).

As a common malignancy of the digestive tract, colorectal cancer (CRC) is ranked second in terms of mortality worldwide and individual treatments are notably various in different patients as a result of personal heterogeneities (Bray et al., 2018; Miller et al., 2019). Serological tests, like enzymes and factors, can easily be completed during the initial screening and monitoring of tumor, but have a considerable false positive and false negative rate. For better clinical benefit, genomics research, as a new direction distinguished from traditional physiology, is being applied in CRC patients to uncover its underlying mechanism for more precise prevention, diagnosis and therapy. Characteristic analysis of CRC patients is an urgent process required to find molecular markers with a high specificity and sensibility.

CSCs in CRC can be isolated from tumor tissues with specific identifications, such as *LGR5*, and have self-renewal and differentiation capacity as stemness (Medema, 2017). Malta et al. (2018) assessed the mRNA expression-based signatures based on the expression values of available stem cell samples with different degrees of stemness from the Progenitor Cell Biology Consortium (PCBC) dataset that compatible with The Cancer Genome Atlas (TCGA) and used one-class logistic regression (OCLR) to derive a predictive model of stemness indices, mRNA expression-based stemness index (mRNAsi), that identified stem cell signatures (Hutter and Zenklusen, 2018). A transcriptomic set of stemness-related epigenetically regulated genes was used to generate the epigenetically regulated-mRNAsi (EREG-mRNAsi) with OCLR that exhibited the methylation/expression relationships and the index showed a positive correlation with both RNA expression and epigenetics. The mRNAsi and EREG-mRNAsi were confirmed to be related with biological processes in CSCs and tumor state for CRC and other tumor samples. These efforts provided data support as a measurement of CSCs for our study.

In this study we aimed to identify which featured genes correlated with CSC stemness based on CRC mRNAsi and EREG-mRNAsi from the results of Malta et al. via bioinformatic analysis. Differentially expressed genes (DEGs) in separate modules were determined through weighted gene co-expression network analysis (WGCNA) (Langfelder and Horvath, 2008). Genes in the significant module were selected for further confirmation and functional analysis. The integrated assessment of key genes associated with stemness in CRC could inform the initiation and progression of tumor and enlighten novel therapeutic target strategies for patients.

## Materials and Methods

### Data Extraction

Expression profiles of RNA-sequencing data from TCGA^1^ and corresponding clinical data were downloaded on January 15, 2020. The stemness indices of CRC patients, including mRNAsi and EREG-mRNAsi, were obtained from the study of Malta et al. (2018). After removal of indices-absent cases, only patients with a definitive diagnosis of CRC and a no less than 30 days overall survival (OS) were brought into the analysis. Furthermore, cases without full clinical parameters on age, sex and Tumor Node Metastasis (TNM) stage were removed from the correlation testing of clinical factors. Finally, 396 CRC patients were included for survival analysis, among these 376 patients had full clinical parameters.

### Survival and Clinical Parameter Correlations Analysis

Survival analyses for the mRNAsi and EREG-mRNAsi were performed on R to identify the prognostic values. The correlations between mRNAsi or and EREG-mRNAsi and the clinical parameters were investigated on the same platform.

### Screening of DEGs

The limma package in R package was used to compare expression levels between normal samples and tumor samples. The criteria for DEG selection were set as follows: fold change (FC) >2 and false discovery rate (FDR) <0.05.

### WGCNA

Genes with the highest 25% of DEG variance were selected to ensure the heterogeneity and accuracy of the bioinformatics statistics for co-expression network analysis. Outliers in RNA-sequencing data were filtered and a Pearson correlation matrix was constructed for the co-expression analysis of paired genes. A weighted adjacency matrix was constructed using the function a_mn_ = | c_mn_| ^b^ (a_mn_ as adjacency between gene *m* and gene *n*; c_mn_ as Pearson’s correlation between gene *m* and gene *n*; b as power showing a strong correlation between genes and penalized the weak correlation). An appropriate b-value was selected to build a co-expression network and the adjacency matrix was converted into a topological overlap matrix (TOM) to measure the connectivity of the genes in the network. Average linkage hierarchical clustering was carried out according to a TOM-based dissimilarity, and the minimum size of the gene dendrogram was 30 for the construction of module dendrograms.

### Featured Gene Identification

The main components were composed of module eigengenes (MEs) and the single expression signature in each given module was summarized by the expression patterns of all genes in the principal components analysis (PCA). Gene significance (GS) was calculated as the log10 conversion of the *P*-value in linear regression between gene expression and mRNAsi or EREG-mRNAsi. Module significance (MS) was calculated as the average GS in a specific module to stand for the correlation between the module and sample traits. A cutoff of less than 0.25 was used to merge similar modules. The correlation between the genes in corresponding modules and gene expression profiles was defined as module membership (MM). After the modules of interest were selected, the GS and MM of each gene were calculated and thresholds for the selection of key genes were set as MM >0.8 and GS >0.5 in modules.

### Differential Expression and Co-expression Analysis

Expression values of the selected genes were compared between the normal samples and tumor samples based on data from the TCGA. The validation utilized mRNA profile in another public database, Oncomine^2^, and the threshold was set as follows: *P*-value, 1E-4; FC, 2; gene level, top 10%.

R package was used to analyze co-expression relationships between selected genes.

### Human Samples

All CRC patients provided written informed consent for research study that was approved by the ethical committees. Tissues including normal mucosae, adenomas, carcinomas and liver metastases were sampled immediately after surgical resection of the specimens from Peking University Third Hospital. The samples were assessed by independent pathologists.

### Immunohistochemical Staining

Tissues (5 μm thick) were deparaffinized and treated with 3% H_2_O_2_-CH_3_OH for 15 min to block endogenous peroxidase. Samples were submerged in pH 6.0 or 9.0 buffer in a pressure cooker for antigen retrieval and then incubated at 37°C for 2 h with antibody *CHEK1* (Abcam, ab40866, 1:400), *BUB1B* (Abcam, ab183496, 1:100), *BUB1* (Abcam, ab195268, 1:100), *TTK* (Proteintech, 10381-1-AP, 1:400), *PLK4* (Proteintech, 12952-1-AP, 1:400), *DNA2* (Proteintech, 18727-1-AP, 1:400), *MCM10* (SAB, 43512, 1:200) and *KIF23* (CUSABIO, CSB-PA23569A0Rb, 1:400). After washing with phosphate buffer saline (PBS), slices were incubated with horseradish peroxidase (HRP)−conjugated IgG (ZSGB−Bio, PV-6000) at room temperature for 30 min and then stained with 3,3N-Diaminobenzidine Tertrahydrochloride (DAB) detection system kit (ZSGB−Bio, ZLI-9018). Protein expression and localization were detected under light microscopy and analyzed by NDP view (version 2.6.8).

### Protein–Protein Interaction (PPI) Network and Functional Enrichment Analysis

A PPI network was constructed on the Search Tool for the Retrieval of Interacting Genes (STRING)^3^.

R package was used to perform gene ontology (GO) analysis for investigation of the biological functions behind selected genes.

### Statistical Analysis

All statistical analyses were performed in R (version 3.6.0)^4^. Wilcoxon testing and Kruskal–Wallis testing were used in the differential analyses. Survival curves were generated through the Kaplan–Meier method and the survival difference of OS was evaluated with log-rank testing. *P* < 0.05 was considered significant in all statistical analyses.

## Results

### Stemness Indices and Clinical Significance in CRC

**FIGURE 1:**
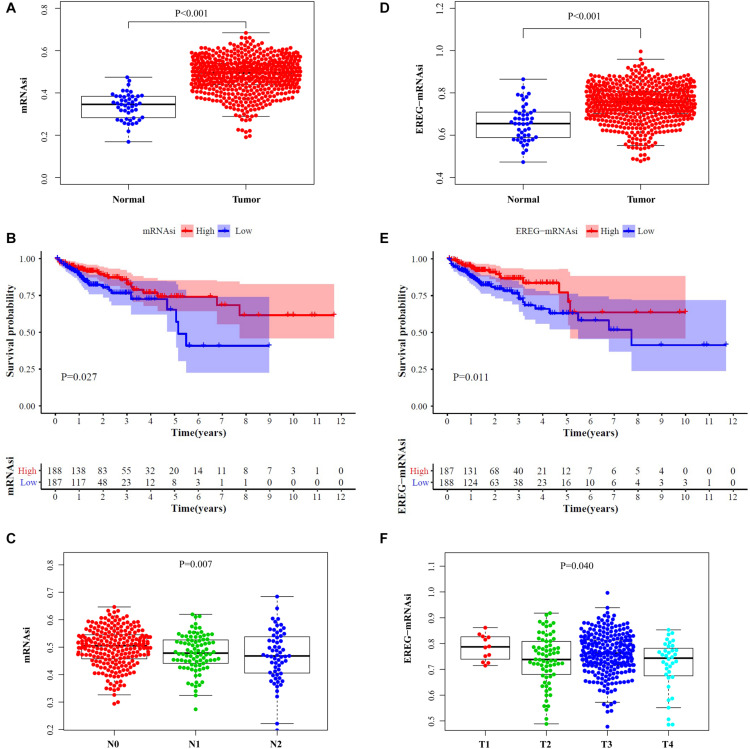
Clinical characteristic analysis. Comparisons of mRNAsi **(A)** and EREG-mRNAsi **(D)** between tumor tissues and normal tissues. Survival curve of mRNAsi **(B)** and EREG-mRNAsi **(E)**. **(C)** The mRNAsi was related to N stage of CRC patients. **(F)** The EREG-mRNAsi was related to T stage of CRC patients.

After comparing the scores of mRNAsi and EREG-mRNAsi between the normal and tumor tissues of CRC patients, we found that mRNAsi and EREG-mRNAsi were both significantly higher in the tumor samples (*P* < 0.001, Figures 1A,D). For investigation of prognostic value, we simultaneously stratified the CRC cohorts into high- and low-score groups on the basis of the medians of mRNAsi and EREG-mRNAsi and constructed survival curves. The high-scores of the mRNAsi and EREG-mRNAsi were obviously associated with a better OS in CRC patients with 5-year survival rates of 0.741 and 0.772, respectively (*P* < 0.05, Figures 1B,E). Age, sex and TNM stage were included for analysis and the results showed that mRNAsi had an overall decreasing trend in N stages, and there was a significant difference in EREG-mRNAsi for different T stages (*P* < 0.05, Figures 1C,F). The two indices were not correlated with age or sex.

### Identification of DEGs and Featured Genes

**FIGURE 2:**
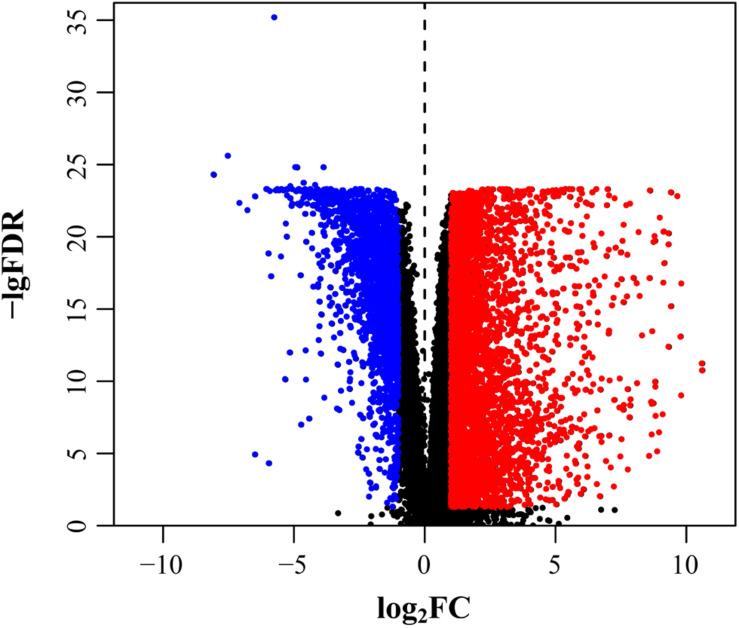
Selection of DEGs. DEGs after screening including upregulation (red) and downregulation (blue).

As mRNAsi and EREG-mRNAsi in tumor samples were significantly different from the normal samples, we screened out stemness-related DEGs based on the CRC indices. After differential analysis, 6,476 DEGs were screened, of which, 4,414 were upregulated and 2,062 were downregulated (Figure 2).

**FIGURE 3:**
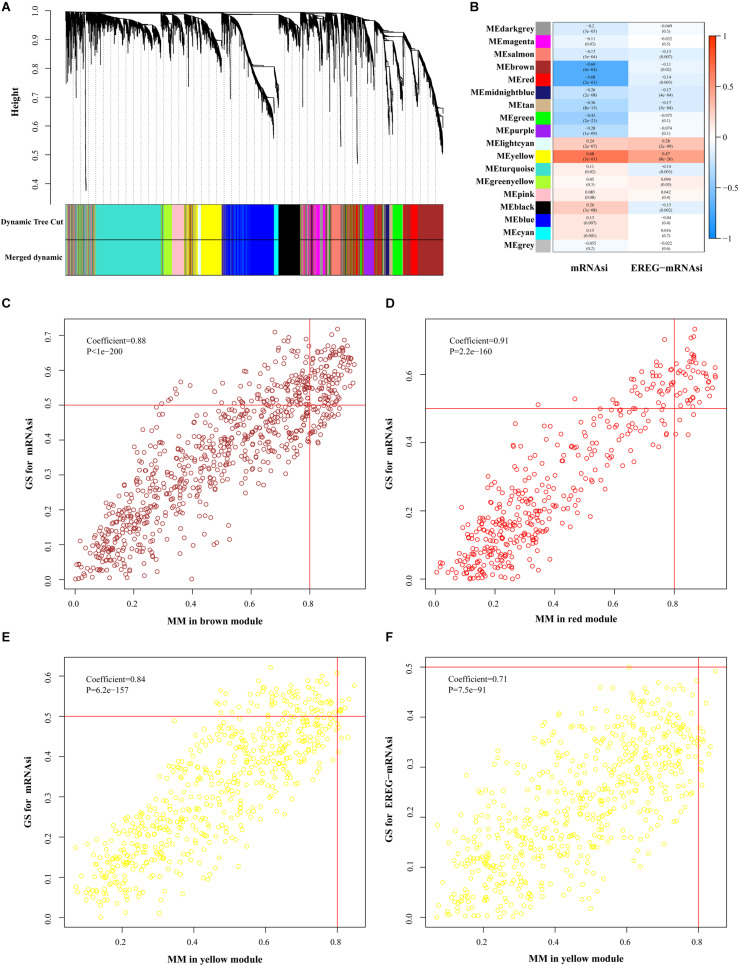
Selection of featured genes. **(A)** Cluster dendrogram in WGCNA that each leave stood for a gene and genes with similar expression pattern composed a branch. **(B)** Correlation between gene modules and indices. The upper row indicated the correlation coefficient and the lower row indicates *P*-value in every module. The criterion was marked in scatter plots including brown **(C)**, red **(D)**, and yellow **(E)** modules of mRNAsi and yellow **(F)** module EREG-mRNAsi. FDR, false discovery rate; FC, fold change; ME, module eigengene; GS, gene significance; MM, module membership.

For further filtering, we used WGCNA to select the DEGs, which assigned genes with similar expression patterns into one module and 36 modules were obtained (Figure 3A). To find correlations between the gene module and indices, we applied MS as the overall gene expression level of the corresponding module. The results showed that the correlations in the brown, red and yellow modules of mRNAsi and the yellow module of EREG-mRNAsi were relatively high, indicating more close associations between genes and indices (*P* < 0.001, Figure 3B). Key genes associated with indices were screened out using the threshold as defined. In the mRNAsi group, 106 genes were in the brown module, 46 genes in the red module and 8 genes in the yellow module, while none of the genes were included in the EREG-mRNAsi module (Figures 3C–F). We selected the yellow module in the mRNAsi group for the subsequent analysis of featured genes (Table 1).

**TABLE 1:** Featured genes after screening.

**Gene**	**Name**
*BUB1*	BUB1 mitotic checkpoint serine/threonine kinase
*BUB1B*	BUB1 mitotic checkpoint serine/threonine kinase B
*CHEK1*	Checkpoint kinase 1
*DNA2*	DNA replication helicase/nuclease 2
*KIF23*	Kinesin family member 23
*MCM10*	Minichromosome maintenance 10 replication initiation factor
*PLK4*	Polo like kinase 4
*TTK*	TTK protein kinase

### Analysis and Validation of Featured Genes

**FIGURE 4:**
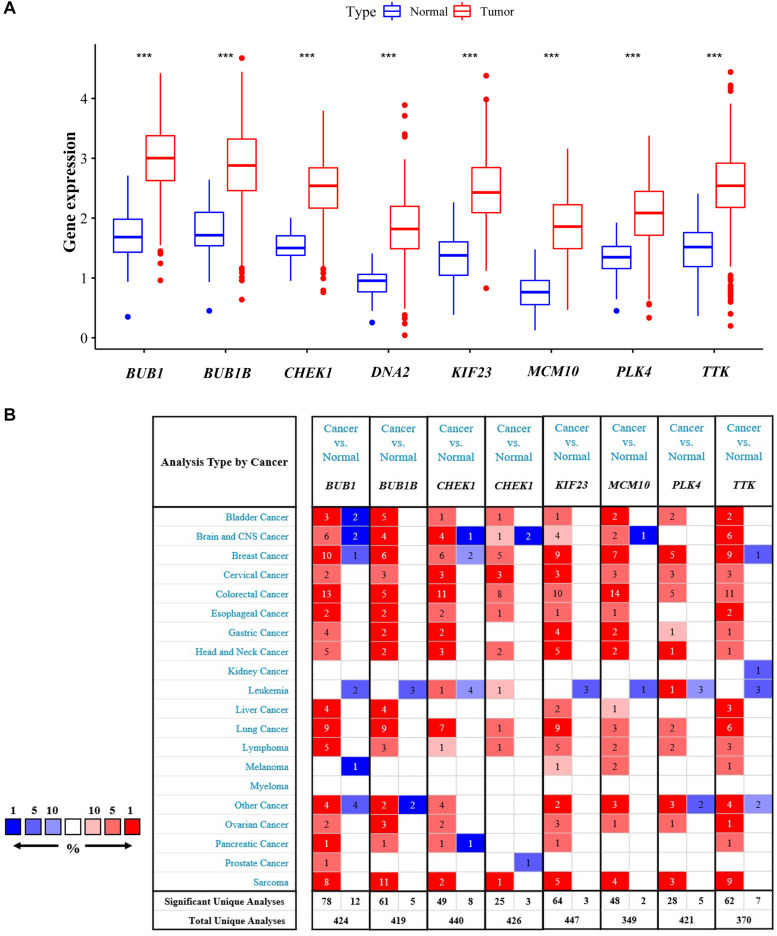
Validation of differential expression in samples. Comparisons of featured genes between tumor tissues and normal tissues based on TCGA **(A)** and Oncomine **(B)**. ***P < 0.01.

Expressions of the featured genes were analyzed with TCGA and Oncomine datasets. Results from the TCGA dataset showed that these genes were indeed obviously overexpressed in CRC tissues (*P* < 0.001, Figure 4A). Additionally, we found that the featured genes were overexpressed not only in CRC but also in many other cancers using the Oncomine dataset (Figure 4B).

**FIGURE 5:**
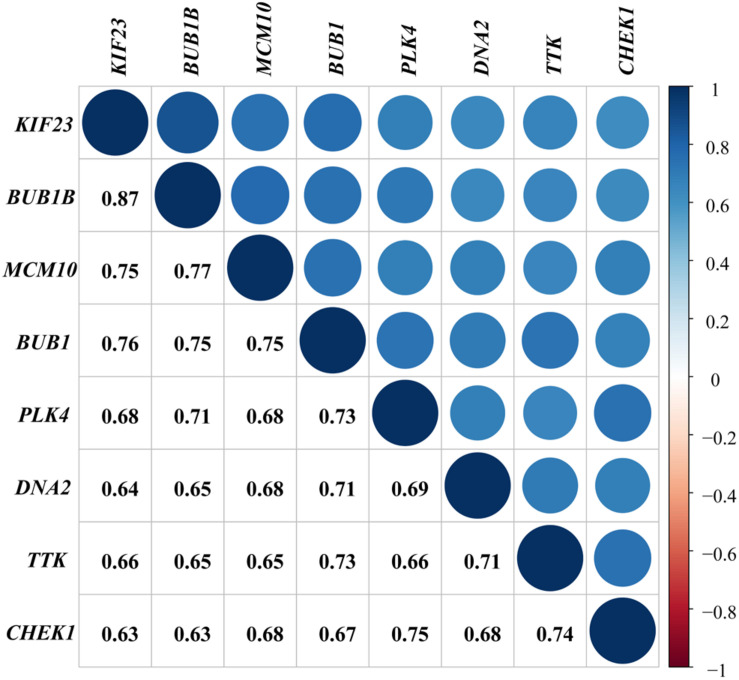
Validation of interrelationship. Co-expression analysis of genes with coefficients annotated.

We performed a co-expression network analysis for further exploration and validated the evident relationship among the featured genes with a coefficient of at least 0.63. The correlation between *BUB1B* and *KIF23* had the highest coefficient of 0.87 (Figure 5).

**FIGURE 6:**
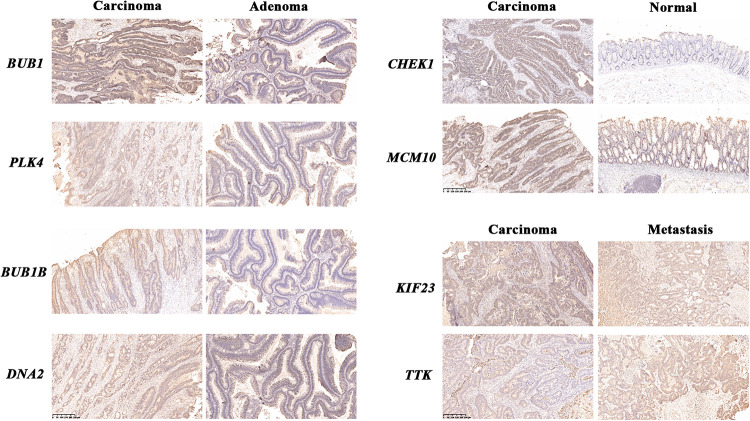
Immunohistochemical staining of human samples. Immunohistochemical staining of normal mucosae, adenomas, carcinomas and liver metastases from CRC patients. The blue stood for nuclear staining and the yellow for target protein staining (scale bars: 250 μm).

For further confirmation at protein levels for these genes, we developed immunohistochemical staining in human samples. Comparing of corresponding adenomas, the expressions of *BUB1*, *BUB1B*, *PLK4*, and *DNA2* in carcinomas were higher. The protein levels of *CHEK1* and *MCM10* were more significant in carcinomas than normal tissues and the differential expressions of *KIF23* and *TTK* could be observed between primary lesions and liver metastases (Figure 6).

### Analysis at Protein Level and Functional Enrichment

**FIGURE 7:**
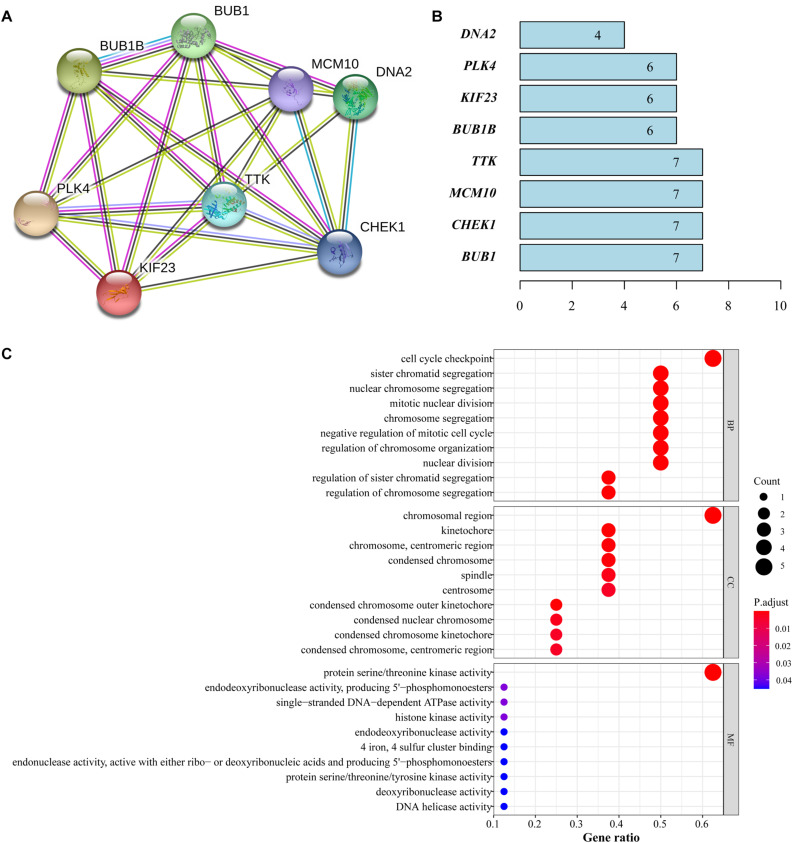
Functional analysis of featured genes. **(A)** PPI network from STRING. **(B)** Node number that each gene correlated. **(C)** GO analysis of featured genes.

We obtained the mutual relationship of the 8 genes at protein level through the STRING and mapped the PPI network, which showed a strong correlation among the featured genes (Figure 7A). Four genes (*BUB1*, *CHEK1*, *MCM10*, and *TTK*) had the highest node number of 7, which indicates that these genes composed a dense interaction in the network (Figure 7B).

Gene ontology analysis was performed and the results revealed that the major biological processes where featured genes are enriched were cell cycle checkpoint, chromosomal region, protein serine/threonine kinase activity and other cell cycle functions closely associated with the proliferation of tumor cells (Figure 7C).

## Discussion

Colorectal cancer exhibits a highly complex tumor heterogeneity in patient populations that are affected by various inter-factors and the mechanisms of oncogenesis and tumor progression need to be explored from different aspects, including microenvironment, metabolism and stemness. The classical theory points out that the initiation of CRC is closely associated with the disorder of stem cells in the crypts, which change into adenoma generation and then canceration (Barker et al., 2009). Normal self-renewal gradually deflects from wild-type stem cells as a result of oncogenic mutations in a long process of accumulation, and genetic and environmental factors play an important role in tumor progression. CSCs remain an increasingly competitive condition and demonstrate advantages over time during tumor evolution, with a confused environment composed of multiple cells, which provide them with different signals (Zeuner et al., 2014). The WNT pathway, Notch pathway and several other signals have been identified as vital factors in the regulation of colorectal CSC (Sikandar et al., 2010; Vermeulen et al., 2010). Precise markers under different classifications used to assess the capacity of stemness are crucial for the translation of CSC discovery into practical clinics and its development for therapy in CRC patients.

Our study focused on the excavation of featured genes that were characterized with stemness in CRC using the proven-effective indices system for in-depth exploration. Based on clinical parameters, mRNAsi was related to lymphatic metastasis and EREG-mRNAsi could reflect tumor infiltration. Both indices showed significant relationships with patient prognosis in accordance with the characteristics of stemness in CRC. WGCNA classified the DEGs with indices and conjugated genes for integral study in a small scale considering the interaction of multiple genes. For the mRNAsi group we obtained optimum genes after filtering, while none of the genes met the threshold in the EREG-mRNAsi group, as clustering with a high correlation was not completed. Although epigenetic regulators were confirmed to be effective in curation targeting at stemness and the EREG-mRNAsi was calculated from a set of stemness-related genes that controlled the epigenome in original study, featured genes about EREG-mRNAsi still failed to summarize under strict criteria (Kreso et al., 2014).

The selected genes were validated to have a strong inner-connection and were tightly associated with the process of tumor progression. *BUB1*, *CHEK1*, *MCM10*, and *TTK* were confirmed to be in the central position of the network. Mutation of *BUB1*, which is known as the spindle assembly checkpoint gene, causes mosaic variegated aneuploidy and gives rise to an increased CRC risk (de Voer et al., 2013). *CHEK1* is a DNA damage sensor at a transcriptional level with the ability to arrest cell cycle for the repairment of damage and participates in defense against evasion. Overexpression of *CHEK1* generally predicts a more serious malignancy and a worse prognosis for CRC patients (Gali-Muhtasib et al., 2008). *MCM10* plays an important role in assembly of complex during DNA replication and its unbalance with other factors broken by DNA damage might contribute to the initiation of cancer (Yoshida and Inoue, 2004). The frameshift mutations of mitotic checkpoint kinase *TTK* in CRC with microsatellite-unstable are frequent and tend to prompt tumor growth because of a triggered alteration in the cell cycle (Ahn et al., 2009). These identified functions have been investigated in other studies and are in accordance with our GO analysis results, illustrating that the set of our featured genes could impact CRC through regulation of the cell cycle and proliferation.

Current evaluations for CRC patients are frequently based on the clinical characteristics such as age, sex and tumor size considering the easy access to data (Colditz et al., 2000; Yeoh et al., 2011). Sequencing technology has drawn gene alteration into disease assessment as precision medicine. Stemness index describes the characteristics of related genes from an independent point of view and is comparatively associated with tumor progression. Our study mined CRC-related biomarkers from this aspect and that is of great significance to be used in clinic. The excavation of stemness index has been reported in other cancer and is proved to have high application value. Pan et al. (2019) identified 13 key genes which played important roles in the maintenance of bladder CSCs in their study. The 12 genes that Pei et al. (2020) found to be closely related in breast cancer stem cell characteristics showed prognosis-oriented effects in patients. Our study in CRC has also acquired 8 meaningful genes and verified by methods of laboratory experiments and bioinformatics.

The featured genes in our study might have several limitations that affect their application and need improvement. Firstly, all of our data came from public databases and further prospective studies need to be implemented to obtain the stemness indices and parameters of CRC patients for the validation of accurate calculation and clinical value. Secondly, the intrinsic association among the featured genes and their relationship with CSC can be experimented on *in vitro* and *in vivo*.

In conclusion, the set of featured genes that we obtained was based on the stemness indices and validated with further analysis. Eight genes were found to be closely related to CSC characteristics in CRC patients and the significant function related to genes was center on the cell cycle, which might support new ideas in individualized preventions and treatments.

## Data Availability Statement

All datasets presented in this study are included in the article/supplementary material.

## Author Contributions

WF designed the study. YL and XZ collected the data. ZL, FL, and WW analyzed and interpreted the data. All authors were involved in writing manuscript and approved of the submitted and published versions.

## Conflict of Interest

The authors declare that the research was conducted in the absence of any commercial or financial relationships that could be construed as a potential conflict of interest.

## Footnotes

1 https://portal.gdc.cancer.gov

2 https://www.oncomine.org

3 https://string-db.org

4 https://www.r-project.org

## Abbreviations

CRC: colorectal cancer
CSC: cancer stem cell
DAB: 3,3N-Diaminobenzidine Tertrahydrochloride
DEG: differentially expressed gene
EREG: epigenetically regulated
FC: fold change
FDR: false discovery rate
GO: gene ontology
GS: gene significance
HRP: horseradish peroxidase
ME: module eigengene
MM: module membership
mRNAsi: mRNA expression-based stemness index
MS: module significance
OCLR: one-class logistic regression
OS: overall survival
PCA: principal components analysis
PBS: phosphate buffer saline
PCBC: Progenitor Cell Biology Consortium
PPI: protein–protein interaction
STRING: Search Tool for the Retrieval of Interacting Genes
TCGA: The Cancer Genome Atlas
TNM: Tumor Node Metastasis
TOM: topological overlap matrix
WGCNA: weighted gene co-expression network analysis.
